# Induced Abortion to Relieve Vomiting in Pregnancy

**Published:** 1885-08

**Authors:** 


### Cullings From Contemporaries.

### INDUCED ABORTION TO RELIEVE VOMITING OF PREGNANCY.

IN view of the fact that a large part of our department of original communications is made up of reports on the use of electricity in vomiting of pregnancy, the following, from the *St. Louis Courier of Medicine*, is extracted as *apropos*, and to give emphasis to the subject. It seems, in the discussion which followed Dr. Scott's report of the case, before the St. Louis Medico-Chirurgical Society, a discussion participated in by Dr. G. A. Moses, Dr. Geo. J. Engelman, Dr. Love, Dr. Scott and Dr. Todd, that the subject of electricity was not mentioned, except incidentally by Dr. Engelman, who, in reporting a case of induced abortion to relieve vomiting—the labor being artificial in all its stages — had used the galvanic current to produce uterine contraction after the *entire contents had been removed.* The articles of Drs. Baird, Williams and Starley are commended to the gentlemen of the St.Louis Medico-Chirurgical Society as well worthy of consideration.

VOMITING OF PREGNANCY—INDUCED ABORTION.

*Dr. Scott:*—On the first of January last a gentleman came to my office and requested me to prescribe for his wife, stating that she was suffering from a severe cold. He thought she was bilious, and that she was a little sick at the stomach. I knew her very well and prescribed for her. The next day he asked me to visit his wife as she was no better. She was still suffering with great nausea, and had some fever. Still thinking that she had taken cold, and was bilious, I visited the lady, and after talking with her about her case, I told her she was pregnant. She had been married on the 14th day of November, and this was the 2nd of January. After examining her very carefully and getting the history of her condition I had no hesitancy in pronouncing her pregnant, and the nausea the result of pregnancy. She was a young woman, and was at first disposed to pass this as a joke, not thinking that she was pregnant.

I then began the treatment of her case of nausea resulting from pregnancy. I gave her everything that the art of medicine could suggest, and everything I gave her was rejected without the least of any of it being retained upon the stomach. Whatever there was in medicine that has been used in such cases or recommended I gave her. Everything that I had read of, or that I had found from my own experience to be beneficial was tried, but I found nothing to be of any service whatever. I used hypodermic injections of morphine, used morphine in the rectum, used it internally, used it in small doses and in large doses; used ipecac in small doses and in large doses, and so I ran through the whole materia medica without producing the least beneficial result upon my patient. I must confess here, that I was beginning to get very much discouraged with the case myself; and upon one occasion her father met me in the parlor and said, "Doctor what is the matter here, and what is going to be the outcome?" I said to him very candidly, "Sir, your daughter is pregnant." "Well, Sir," says he, "What is going to be the outcome?" I said, "I have tried everything I can suggest, and there is but one thing to be done, and that is to produce a miscarriage. That is the only thing that will give her any relief whatever." I might say I had dilated the os, I had touched it with iodine, I had touched it with nitrate of silver, and nothing produced the least beneficial results. I told him that it was the last resort, and that I had been trying everything for her within the last two weeks, hoping that with the growth of the fetus this nausea would cease. This was one of the most distressing cases I ever saw. I sat at her bedside for twelve and twenty-four hours; and I don't believe that there was an interval of fifteen minutes that there was not vomiting. She vomited whatever I gave her, as a matter of course. The father felt a little hesitancy in submitting to such a proposition, but I told him it was the only resort; but, said I, "If you want a consultation, if you want any one else in this matter, have any one you please to see her. Accordingly Dr. Moses and I saw the case together. At first he didn't agree with me as regards her pregnancy. On making a vaginal examination he said that there was parametritis and some inflammation, and that this was probably the cause of the nausea. But at his next examination he was satisfied that she was certainly pregnant. There was some parametritis, which was evidenced by the fixedness of the uterus. She was just married and had taken a long bridal trip, and submitting for the first time to the approaches of her husband, there necessarily resulted some slight parametritis.

I had known the patient from childhood, and she had perfect health; she was married November 14th; coming back from a long bridal trip with nausea, with constant sick stomach, it was quite natural to suppose her pregnant. Her head was clear; there was no trouble with the lungs; the kidneys were in perfect condition, I examined her urine; her bowels moved regularly. Now the question was, "What could cause this nausea except pregnancy?" And this was precisely my ground for my arriving at the conclusion which I did, and this reasoning convinced Dr. Moses, on our second meeting, of the correctness of my conclusion.

I told Dr. Moses in detail what I had been doing, and then we treated her together for a week or ten days; and we could produce no good result. I told Dr. Moses, when he first came to see her, that an abortion must be produced, that it was the only way in which we could produce any good result. The family were exceedingly uneasy about the lady, and Dr. Boisliniere was also called in consultation. He, however, would not consent to an abortion. He proposed to me to try something else and keep trying. He said he had a good many of these cases when almost on the verge of the grave recover, and suggested some of the very same things I had used, and many others I had not used. The use of belladonna plaster over her uterus, and belladonna suppositories—the belladonna suppositories I had been using. Nothing at all had any favorable effect, so that Dr. Boisliniere finally reluctantly gave his consent to the abortion. Dr. Moses introduced the sound into the uterus and thought he broke up the membranes, but we waited all that day and all that night, and no results whatever followed even the introduction of the sound. I still adhered to my view of the case that she was pregnant and insisted on it, and insisted that we must do this thing over again, in which Dr. Moses agreed with me. The second day after the first trial, Dr. Moses again introduced the sound, using Sims' speculum. This was done about ten or eleven o'clock. Again there was no sufficient indication of labor, nausea continuing all the time; and about two o'clock in the morning I had the napkins removed, and I found a little stain of blood upon them. I told her mother that she might lie down and rest easily, that it would soon be over. At seven o'clock, on removing the napkins we found the fetus. I don't think she vomited twice afterwards. I afterwards removed the secundines and the patient made a good recovery.

It was on the 26th of January that the abortion was produced. From the very time that the fetus came away her nausea left her. I was satisfied that this was the only thing which would be of any good. This was the third case I have had within eighteen months in which a similar condition of affairs existed,and when I produced an abortion the patients were saved. I felt that I was treading ground that I had gone over before.

